# Antiretroviral‐Induced Toxicity in Umbilical Cord Blood‐Derived Haematopoietic Stem/Progenitor Cells

**DOI:** 10.1111/jcmm.70557

**Published:** 2025-04-27

**Authors:** Candice Laverne Hendricks, Andrea Ellero, Juanita Mellet, Voula Stivaktas, Michael Sean Pepper

**Affiliations:** ^1^ Department of Immunology, Faculty of Health Sciences, Institute for Cellular and Molecular Medicine University of Pretoria Pretoria South Africa; ^2^ SAMRC Extramural Unit for Stem Cell Research and Therapy, Faculty of Health Sciences University of Pretoria Pretoria South Africa; ^3^ Department of Pharmacology, Faculty of Health Sciences University of Pretoria Pretoria South Africa; ^4^ Beckman Coulter Life Sciences Pretoria South Africa

**Keywords:** antiretroviral drugs, ARV toxicity, colony forming units, haematopoietic stem cells, HIV‐exposed uninfected, immunophenotype, in utero ARV exposure, umbilical cord blood haematopoietic stem cells

## Abstract

Improvements in administration and efficacy of antiretroviral therapy (ART) have reduced rates of mother‐to‐child transmission (MTCT) of human immunodeficiency virus (HIV) to < 2%. However, in utero exposure to antiretrovirals (ARVs) leads to abnormalities in HIV‐exposed uninfected (HEU) infants. We determined the effect of five ARVs on human umbilical cord blood (UCB)‐derived haematopoietic stem/progenitor cells (HSPC) with the aim of exploring a potential causal relationship with haematological abnormalities. Efavirenz (EFV) was cytotoxic to HSPCs alone and in combination with tenofovir disoproxil fumarate (TDF) and emtricitabine (FTC). Dolutegravir (DTG) had a biphasic effect on HSPC expansion. Immunophenotypic analysis showed increased erythroid and granulocyte precursors after 7‐day culture with these drugs. Finally, using colony forming unit (CFU) assays, we observed impairment in the formation of CFU‐GEMM and CFU/Burst forming unit (BFU) erythroid (E) in the presence of DTG, lamivudine (3TC) and TDF, both visually and immunophenotypically. We conclude that multiple potential HSPC toxicities exist with ARVs commonly used in pregnancy, prompting the need for further research to confirm the safety of these drugs in this vulnerable group.

## 1 Introduction

Of the 39 million people living with human immunodeficiency virus (HIV) (PLWH) globally, the highest prevalence is in sub‐Saharan Africa [1]. The evolution of antiretroviral (ARV) therapy (ART) used to treat HIV since the initial introduction of the first nucleoside reverse transcriptase inhibitor (NRTI), Zidovudine (AZT) in 1987 [2, 3], has resulted in the development of five classes of drugs with a combined number exceeding 25 [2, 4]. The advent of improved life expectancy, driven by the global implementation of ART regardless of CD4‐T lymphocyte count [5, 6], has raised concerns about potential toxicities associated with long‐term use of these drugs [7]. Pregnant women LWH have the added concern of exposure of the fetus in utero to ARVs. ARVs not only control HIV in the mother, but also prevent HIV transmission to the fetus, as ARVs have been shown to cross the placenta efficiently [8, 9].

For most NRTIs and non‐nucleoside reverse transcriptase inhibitors (NNRTI), transplacental transfer is relatively consistent with similar exposures documented between maternal and cord blood serum at a cord blood to maternal serum (C:M) ratio of 0.6–1.6 [8, 9]. Protease inhibitors and Efavirenz (EFV) have poorer penetration with C:M ratios of < 0.6 [8, 9] and 0.5–0.7 [10, 11], respectively. Dolutegravir (DTG), an integrase strand transfer inhibitor (integrase inhibitor) and one of the newer ARVs, is part of the fixed‐drug combination (FDC) tablet used as first line ART in South Africa [12], and it is also recommended for first line use globally by the World Health Organisation (WHO) [13]. DTG has been shown to be a more potent suppressor of viral load compared to EFV [14, 15], driving its popularity.

The success of ARVs has led to > 98% of infants born to women LWH in South Africa being HIV uninfected [16], as occurs in many parts of the world [17, 18]. These HIV‐negative infants exposed to maternal HIV [HIV exposed uninfected (HEU) infants] are, however, not spared drug‐related toxicities as a result of ARV exposure. HEU infants have poor growth trajectories [19], delayed neurodevelopment [20, 21], and immune dysregulation [22, 23]. Within the haematological system, anaemia with or without neutropenia [24, 25, 26, 27, 28] has been described extensively, and the ARV most often implicated is AZT. Nielsen et al. [29] observed decreased CD34+ haematopoietic progenitor colony forming unit (CFU) capacity in infants exposed to AZT, despite CD34+ cell numbers being equivalent to their HIV unexposed uninfected (HUU) counterparts. AZT is still used in neonatal ARV regimens [12, 30], although it is now only used under special circumstances in adults [13].

The direct impact of ARVs on haematopoietic stem and progenitor cell (HSPC) immunophenotype in vitro has not been determined, and data on the influence of ARVs on CFU formation is limited. These data could add to our understanding of the pathophysiology of haematological abnormalities in HEU infants. In this study, freshly isolated umbilical cord blood (UCB)‐derived CD34+ HSPCs from HEU infants were exposed in vitro to ARVs used in fixed‐drug combination (FDC) tablets in South African ARV regimens, namely, TLD [Tenofovir disoproxil fumarate (TDF), Lamivudine (3TC), DTG] and TEE [TDF, Emtricitabine (FTC), EFV]. Given the effect of the high degree of genetic diversity in South Africa's population on drug metabolism [31], a wide concentration range was used for each drug. The purpose was to obtain insight into the possible mechanisms of haematological toxicity in HEU infants outside of the AZT era.

## 2 Methods

### 2.1 Drug Reconstitution and Determination of Concentrations

TDF, 3TC, and EFV (20 mg each) were obtained from the National Institutes of Health (NIH) HIV Reagent Program (Virginia, USA); DTG and FTC were obtained from Abcam (Cambridge, UK) at 5 mg and 50 mg, respectively. Drugs were reconstituted in dimethyl sulfoxide (DMSO) to a concentration of 20 mg/mL, except for DTG which was at 5 mg/mL. Concentrations for experiments were based on previously described relative maximal plasma concentration (*C*_max_) for each drug: TDF = 0.296 μg/mL [32], 3TC = 2.04 μg/mL [33], DTG = 3.67 μg/mL [33, 34], FTC = 1.8 μg/mL [32, 35], and EFV = 4.1 μg/mL [36]. The decision to use the *C*_max_ was based on reports of maternal to cord blood (M:C) ratios [8, 9, 10, 11, 37].

After initial reconstitution, working stock solutions were prepared in StemSpan animal origin free (AOF) medium (StemCell technologies, Canada) in 2 mL sterile Eppendorf tubes. Dose responses for each drug used a half‐log dilution series from 20‐fold (20×) of each *C*_max_ down to 0.2× *C*_max_. For DTG, the in‐well DMSO concentration was noted to be > 1% for the 20× *C*_max_ starting point, due to solubility saturation; however, the maximal vehicle concentration for all other drugs was < 0.5%. Subsequently, dose responses for the clinical combinations, i.e., TLD and TEE, were assessed. To avoid excessive toxicity, as informed by the individual dose responses, concentrations started at 5× *C*_max_ and were diluted (half‐log) down to 0.01×. Finally, cells were exposed to drugs in their respective clinical combinations (TLD and TEE) during cell culture and CFU experiments. All drugs, except EFV (0.5× *C*_max_), were administered at *C*_max_.

### 2.2 Sample Collection and Processing

Pregnant HIV‐negative mothers undergoing elective caesarean section at term (37–42 weeks gestation) at public and private hospitals in Pretoria, South Africa, who provided informed consent, were included in the study. All UCB samples tested negative for HIV using the GeneXpert 1 System (Cepheid, USA) and HIV‐1 Qual XC cartridges (Cepheid, USA). Processing of UCB occurred within 24 h of collection (stored at 4°C). Blood was layered onto a density gradient medium, Histopaque‐1077 (Sigma‐Aldrich, USA), in sterile 50 mL Falcon tubes and centrifuged for 30 min at 1700 rounds per minute (rpm). The buffy coat was removed and washed with TP buffer [phosphate buffered saline (PBS) pH 7.4, 10 μg/mL human albumin (Sigma‐Aldrich) and 2 mM ethylenediaminetetraacetic acid (EDTA)], after which red blood cells (RBCs) were lysed with ammonium chloride solution. After further processing, two 100 μL aliquots were stained for counting, one with Stem‐Kit (Stem‐Kit HSPC enumeration kit, Beckman Coulter, Miami, USA) CD45‐isotype [CD45 (clone J33) FITC and IsoClonic control PE] and the viability dye 7‐AAD, and the other with Stem‐Kit CD45/CD34 [CD45 (clone J33) FITC and CD34 (clone 581) PE and 7‐AAD].

### 2.3 HSPC Enrichment

Following determination of the total mononuclear cell (MNC) count, cells were enriched for CD34+ HSPCs according to the Miltenyi CD34 MicroBead Kit instruction manual (Miltenyi Biotec, Germany). CD34+ cell purity was ≥ 95% prior to cell culture experiments for both the initial cell cytotoxicity and cell viability assays, as well as for the immunophenotyping and CFU experiments.

Due to the high CD34+ cell number required and the relatively low and variable number of CD34+ cells available for seeding from each biological replicate on day 0 (D0), each experimental subset (acute and chronic individual and combination drug cytotoxicity experiments; combination drug experiments for 7‐day culture and 14‐day CFU) was performed on at least 3 biological replicates, with each replicate derived from either a single donor (combination drug experiments) or two‐donor pools (individual drug experiments) contingent on cell numbers post isolation (19 unique donor samples total).

### 2.4 HSPC Culture for ARV Viability Assays

After enrichment, cells were plated at 2 × 10^3^ cells/well in 96‐well plates. ARV exposure was assessed as acute (24 h) or chronic (7‐day) for both individual and combination drug experiments.

Cell suspensions were made up in StemSpan AOF medium supplemented with 2% pen/strep [100 units/mL penicillin (GibcoBRL) and 0.1 mg/mL streptomycin (GibcoBRL)] as well as growth factors: stem cell factor (SCF), thrombopoietin (TPO), FMS‐like tyrosine kinase 3 ligand (FLT3L), granulocyte colony‐stimulating factor (G‐CSF), and interleukin‐3 (IL‐3) (Life Technologies, Thermo Fisher Scientific, USA), each at 100 ng/mL. For individual drug exposure experiments, each ARV was added in triplicate at six concentrations (0.2× *C*_max_, 0.63× *C*_max_, *C*_max_, 2× *C*_max_, 6.3× *C*_max_, 20× *C*_max_).

For acute drug exposure experiments, cells were seeded on D0 and expanded for 6 days, followed by exposure to the drugs for 24 h. Expansion was required in order to obtain a sufficient number of cells for accurate assessment of drug effect. For chronic exposure, cells were seeded together with drugs on day 0 and cultured in the presence of drugs for 7 days. All plates had a blank, negative control, positive control, and a range of vehicle control (VC) exposures to rule out any potential non‐drug‐related influence on viability (0.0625%–0.5% DMSO).

Potential cytotoxicity was measured on day 7 (D7) using the lactate dehydrogenase (LDH) assay following a modified version of the manufacturer's protocol. Briefly, the reaction mixture was prepared by combining 11.4 mL of assay substrate, solubilised in distilled water, with 0.6 mL assay buffer under conditions of light exclusion. Positive (LDH Max) controls were prepared by collecting three wells worth of HSPCs into an Eppendorf tube and adding 10× lysis buffer. Lysis was further aided by vortexing. HSPCs were collected to the bottom of the wells by centrifugation (800 *g*, 5 min), and 50 μL of medium was transferred into new 96‐well plates along with 50 μL from LDH Max, vehicle, negative, and blank controls. Subsequently, 50 μL of the reaction mixture was added to each well, incubated for 45 min, and quenched by the addition of 50 μL of stop solution. Absorbance was recorded at 490 nm using a Bio‐Rad iMark spectrophotometer (Bio‐Rad, South Africa). Cellular metabolic activity as a secondary inference for viability was measured using the resazurin assay. A 100x stock solution was prepared by dissolving 250 mg resazurin into 50 mL sterile PBS (pH 7.4), which was further diluted to a 4× working solution. Following supernatant removal for LDH as described above, 50 μL of resazurin working solution was added to the remaining cell solution and incubated for 24 h before transferring 100 μL to a clear bottom black plate. Conversion of resazurin to resorufin was measured by fluorescence at excitation and emission wavelengths of 560 and 590 nm, respectively, using a BMG labtech FLUOstar Omega fluorometer (BMG labtech, Germany).

For combination drug experiments, TLD and TEE were used and plated in 96‐well plates at 6 concentrations (0.01× *C*_max_, 0.032× *C*_max_, 0.1× *C*_max_, 0.32× *C*_max_, *C*_max_, 5× *C*_max_) based on the results of individual drug experiments. These concentrations were applied to each drug within the respective combinations. To account for differences in pharmacokinetic profiles not typically reflected in commercial preparations, the combination drug concentrations were determined ratiometrically, with each drug's concentration adjusted according to its specific *C*_max_ value (all drugs excluding EFV [0.5x *C*_max_] due to toxicity limitations), as described previously. All acute and chronic drug experiments were completed in batches of at least three (*n* = 3) biological replicates in triplicate.

### 2.5 HSPC Culture Plating for Expansion Experiments

After the individual and combination experiments had provided insight into innate and drug exposure related toxicity, cell culture experiments were undertaken on a different set of biological samples. Cells were cultured for 7 days at higher cell numbers (1 × 10^4^ cells/well in 24‐well plates in triplicate, with VC) to determine the effect of combination drugs (TEE and TLD) on the total cell number and phenotype on D7. Cells were exposed to drug concentrations corresponding to *C*_max_, except for EFV (exposure at 0.5 *C*_max_, informed by LDH and resazurin assays). After 7 days in culture, cell number was assessed using the Stem‐Kit HSPC enumeration kit (Beckman Coulter, Miami, USA) and immunophenotypic analysis was performed.

### 2.6 Immunophenotypic Analysis

Immunophenotype was determined on D0 and D7; however, comparative analysis was undertaken on D7 only, after cells had been exposed to drugs. Differences between D0 and D7 immunophenotype of control Lin‐CD34+ cells have been extensively discussed in a recent publication by our group [38, 39] and are not the focus of this manuscript. An aliquot of between 5 × 10^5^ and 1 × 10^6^ total MNCs was removed to be stained for immunophenotypic analysis using the following anti‐human monoclonal antibodies: Lin‐FITC [clones: CD3, (UCHT1); CD14, (HCD14); CD16, (3G8); CD19, (HIB19); CD20, (2H7); CD56, (HCD56); BioLegend, San Diego, USA], CD34‐APC‐AF700 (clone: 581; Beckman Coulter, Miami, USA), CD38‐ECD (clone: LS198‐4‐3; Beckman Coulter, Miami, USA), CD133‐PE‐Violet770 (clone: REA753, Miltenyi Biotec, Germany), CD117‐PE (clone: 104D2; BioLegend, San Diego, USA), CD90‐BV510 (clone: 5E10; BioLegend, San Diego, USA), CD45RA‐APC (clone: 2H4; Beckman Coulter, Miami, USA), and CD49f‐SB780 (clone: G0H3; BioLegend). Cells were incubated with antibodies for 15 min, and the viability dye zombie violet (ZV) was added (incubation 15 min) prior to washing and analysis. The volume of antibody added was determined through antibody titrations, using the stain index [Stain Index = (Median of Positive—Median of Negative) / (Standard deviation of Negative × 2)] to assess optimal staining intensity. Samples were analysed using a CytoFLEX flow cytometer (Beckman Coulter, Miami, USA).

### 2.7 Colony Forming Unit Assay andImmunophenotype

CFU plates were prepared by plating 200 CD34+ HSPCs/well (in 4 μL) in MethoCult semi‐solid medium (up to 500 μL/well) with either TLD or TEE drug combinations, each in triplicate, in a 24‐well plate. Each drug within TLD and TEE was added to the cell suspension at 10 μL per drug except EFV which was added at 5 μL. Volumes of drugs (total 25–30 μL per well) and cell suspension (4 μL per well) adhered to the manufacturer's recommended drug‐to‐culture medium ratio of no more than 10% [40]. Cell and drug suspensions were cultured for 14 days in 5% CO_2_ at 37°C. CFU counting was informed by the CFU atlas containing images from “The Human Colony Forming Cell (CFC) Assay Using Methylcellulose‐based Media” protocol (R&D Systems, USA) [41]. CFU‐granulocyte, erythroid, macrophage, megakaryocyte (GEMM), CFU‐granulocyte‐macrophage (CFU‐GM), granulocytes (CFU‐G), macrophages (CFU‐M), and erythrocytes (B/CFU‐E) colonies were identified. After counting the colonies, PBS was added to all wells and the cell suspensions with each drug combination were transferred in their individual combinations (TLD and TEE) to 15 mL Falcon tubes. Suspensions were centrifuged at 3000 × *g* for 10 min and resuspended in 200 μL PBS. A 100 μL aliquot was stained with the following antibodies: CD235a‐PE (clone: 11E4B‐7‐6), CD71‐APC‐AF750 (clone: YDJ1.2.2), CD41‐ECD (clone: P2), CD15‐Krome Orange (clone: 80H5), CD14‐APC‐AF700 (clone: RM052), CD19‐PC5.5 (clone: J3‐119), CD20‐PC5.5 (clone: B9E9), CD3‐PC5.5 (clone: UCHt1), CD56‐PC5.5 (clone: NP01), and CD33‐APC (clone: D3HL60.251) [All Beckman Coulter (Beckman Coulter, Miami, USA)]. The second 100 μL aliquot was stained with the viability dye, 7‐AAD. Post incubation and washing, data was acquired on a CytoFLEX flow cytometer. The gates used to determine viability were extrapolated to the immunophenotype tube to allow for the inclusion of viable cells only in the immunophenotypic analysis.

### 2.8 Data Analysis

Data from LDH and resazurin assays were exported into Microsoft Excel spreadsheets for processing. The data was blank‐adjusted before further processing. Spontaneous LDH release was accounted for by subtracting the control LDH release from each experimental condition. Percentage cytotoxicity compared to max was calculated using the formula below, where experimental LDH release is the baseline‐corrected LDH release per experimental condition and LDH Max is the baseline‐corrected LDH Max control.

$\%\text{Cytotoxicity}=\frac{\;\text{Experimental}\;\mathrm{LDH}\;\text{release}\;\left(490\;\mathrm{nm}\right)}{\mathrm{LDH}\;\mathrm{Max}\;\text{release}\;\left(490\;\mathrm{nm}\right)}\times 100$

Cytotoxicity values below zero were imputed as 0. Data matrices were then imported into R Studio for statistical processing using the ggstats package, and significance was determined using the Kruskal‐Wallis and Dunn's tests for pairwise comparisons. For resazurin, metabolic activity (as an inference for cell viability) was expressed as a percentage relative to non‐drug‐exposed controls. Data matrices were imported into GraphPad Prism for analysis, and where possible, half‐maximal inhibitory concentration (IC_50_) values were calculated using three‐parameter normalised non‐linear regression. Mean relative viability per dose/condition was imported into R Studio, and summary heatmaps were generated using the pheatmap package (DOI: 10.32614/CRAN.package.pheatmap).

Count data for total and CD34+ cell populations were log2 transformed and visualised in R Studio using the ggstats package, and significance was determined using Fisher's F‐test and Tukey's HSD for pairwise comparisons. For expansion and CFU immunophenotypic analysis, a limited gating strategy was performed in Kaluza Analysis Software (version 2.1; Beckman Coulter, Miami, USA) before being imported into the Cytobank platform (http://www.cytobank.org; Beckman Coulter, Miami, USA). Peak Extraction and Cleaning Oriented Quality Control (PeacoQC) removed anomalous events, and a CD34/Lin bivariate plot identified the Lin‐CD34+ population from which further analysis was undertaken. Dimensionality Reduction (DR) was performed using Uniform Manifold Approximation and Projection (UMAP) plots with an equal number of Lin‐CD34+ events per condition used for the analysis. After DR, a flow cytometry self‐organising map (FlowSOM) clustering algorithm was applied to the UMAP to identify specific clusters in an unbiased manner. Once the clusters were identified, they served as a template from which manual gating of the UMAP was undertaken (see Figure S2A). The manual gates were then assigned population names based on the immunophenotype identified within the gate (Figure 3C). The populations were named according to existing nomenclature for HSPC subsets [42, 43, 44], but the term ‘extended’ was added as a prefix. This was required in order to avoid confusion with what is known in the literature and to distinguish our sub‐populations which have not previously been described with this combination of markers. The phenotype nomenclature for these populations has also been included in other published [38] manuscripts from our group. FlowSOMs also determined statistically significant differences in populations in the D7 and D14 subsets using box plots, bar graphs, and heat maps. Statistical analysis was performed using a non‐parametric Kruskal–Wallis test on individual files within the Cytobank platform. Grouped analyses for each metacluster (MC) were conducted using GraphPad Prism software (v8.0.1) with multiple comparison t‐tests to assess differences between control and treatment groups.

## 3 Results

**FIGURE 1:**
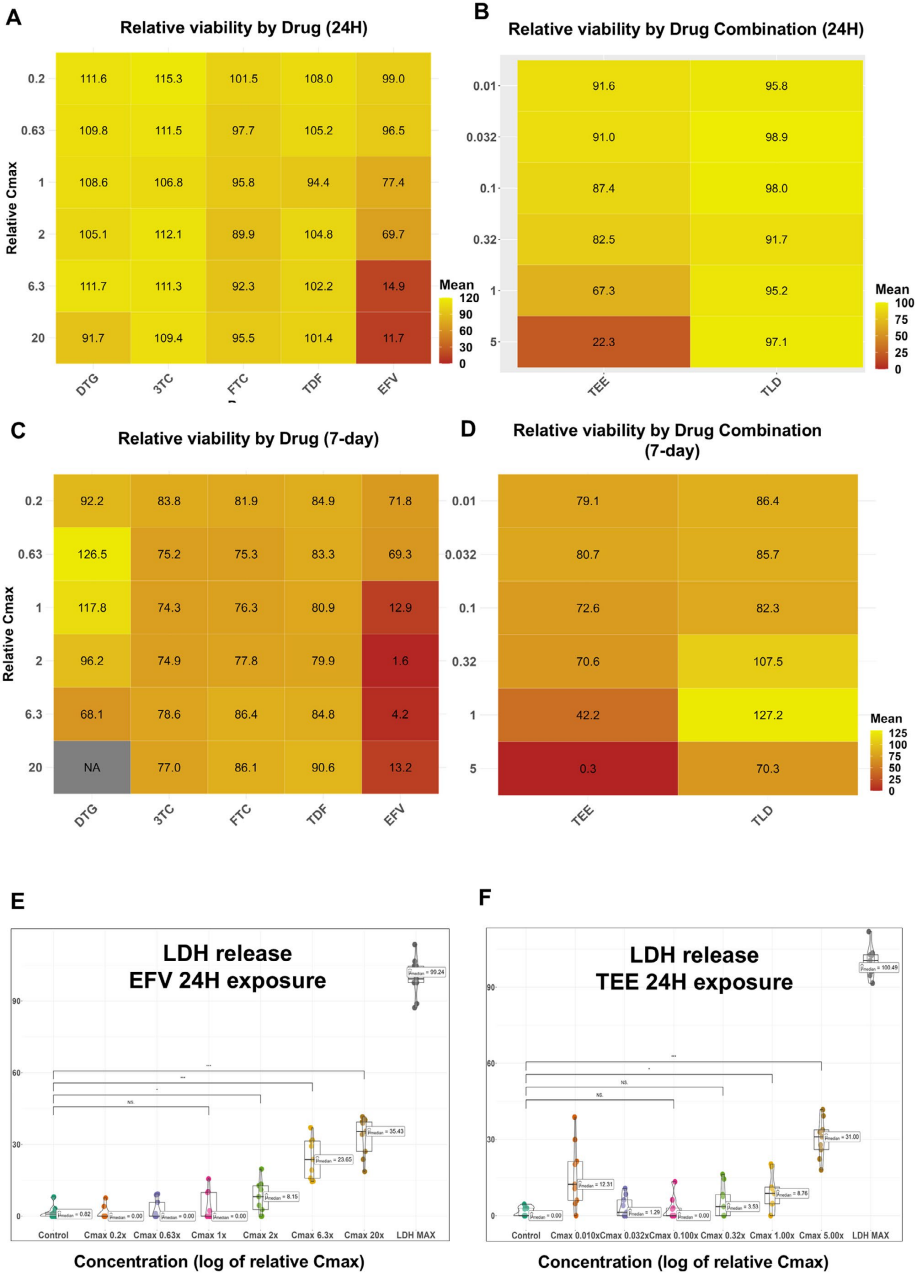
Resazurin and LDH assays indicating cell viability and cytotoxicity following exposure to drugs and drug combinations. (A, B) Heat maps indicating viability as measured using the resazurin assay after 24‐hour (24H) exposure to individual and combination drugs, expressed as percentage relative to the vehicle control (VC) for ARVs as either single agents (3TC = lamivudine, DTG = dolutegravir, EFV = efavirenz, FTC = emtricitabine, TDF = tenofovir disoproxil fumarate) (A) or in clinically approved combinations (TEE = efavirenz/emtricitabine/tenofovir disoproxil fumarate, TLD = tenofovir disoproxil fumarate/lamivudine/dolutegravir) (B). Drug is shown on the x‐axis, concentration relative to *C*_max_ is on the y‐axis. Colour scale of viability is on the right of each figure. Half‐maximal inhibitory concentrations (IC_50_) were only achieved in EFV or EFV‐containing combinations at approximately 1.67× *C*_max_ for TEE and 2.81× *C*_max_ for single agent EFV. (C, D) Similar heat maps as displayed in A and B, showing the resazurin assay performed after 8 days of exposure to drugs and drug combinations. EFV and TEE substantially impacted cell viability with 7‐day IC_50_ values of 0.72 and 0.49× *C*_max_ for EFV and TEE, respectively. An increase in cell viability was observed with DTG at 0.63× *C*_max_ and *C*_max_. The same trend was observed for TLD at 0.32× *C*_max_ and *C*_max_. (E, F) Violin with nested box and whisker plots for EFV (E) and TEE (F) showing multiplexed LDH release after 24‐h exposure to various concentrations (x‐axis) as percentage relative to maximum control (y‐axis). No increase in LDH release was found for TDF, 3TC, DTG, or FTC or TLD (data not shown). EFV and TEE were cytotoxic to cells within 24 h, with an increasing trend of LDH release corresponding with increasing drug concentration, with statistically significant (*p < 0.05 and ***p < 0.001 increases being noted from concentrations>*C*_max_ for EFV [*n* = 3 for EFV and *n* = 3 for TEE]). See also Figure S1B,C.

After acute exposure to TDF, 3TC, DTG, and FTC, the resazurin assay revealed that when compared to controls, viability was not reduced (Figure 1A). For EFV, however, a concentration‐dependent decrease in cell viability was seen within 24 h, with an interpolated IC_50_ of 2.81× *C*_max_ (Figure 1A) Similarly, using the combinations, exposure to TLD for 24 h had no effect, whilst a significant reduction in cell viability was noted following TEE exposure at both *C*_max_ and 5× *C*_max_, with an IC_50_ of 1.67× *C*_max_ (Figure 1B). After 8 days in culture (D8), the resazurin assay revealed a minor concentration‐independent reduction in cell viability (70%–90%) for TDF, 3TC, and FTC relative to controls (Figure 1C). However, in the presence of EFV, a sharp, dose‐dependent decline in cell viability was noted at concentrations exceeding 0.63× *C*_max_ (Figure 1C) Interestingly, a seemingly increased viability was observed with DTG exposure at two concentrations (0.63× *C*_max_ and *C*_max_) when compared to controls (Figure 1C). IC_50_ values were attainable for EFV exposures only, with interpolated IC_50_ values of 0.72× *C*_max_. The same trend that was observed with DTG (Figure 1C) was observed with TLD (which contains DTG) after 8 days in culture, with an increase in cell viability at certain concentrations of TLD (0.32× *C*_max_ and *C*_max_) (Figure 1D). For TEE, viability was decreased in a dose‐dependent manner with an interpolated IC_50_ value of 0.49× *C*_max_ (Figure 1D).

LDH release was not increased following acute exposure (24 h) (Figure S1A–D), except in the case of EFV (Figure 1E) and TEE (Figure 1F), both of which showed a trend toward increased LDH release with increased drug concentration.

**FIGURE 2:**
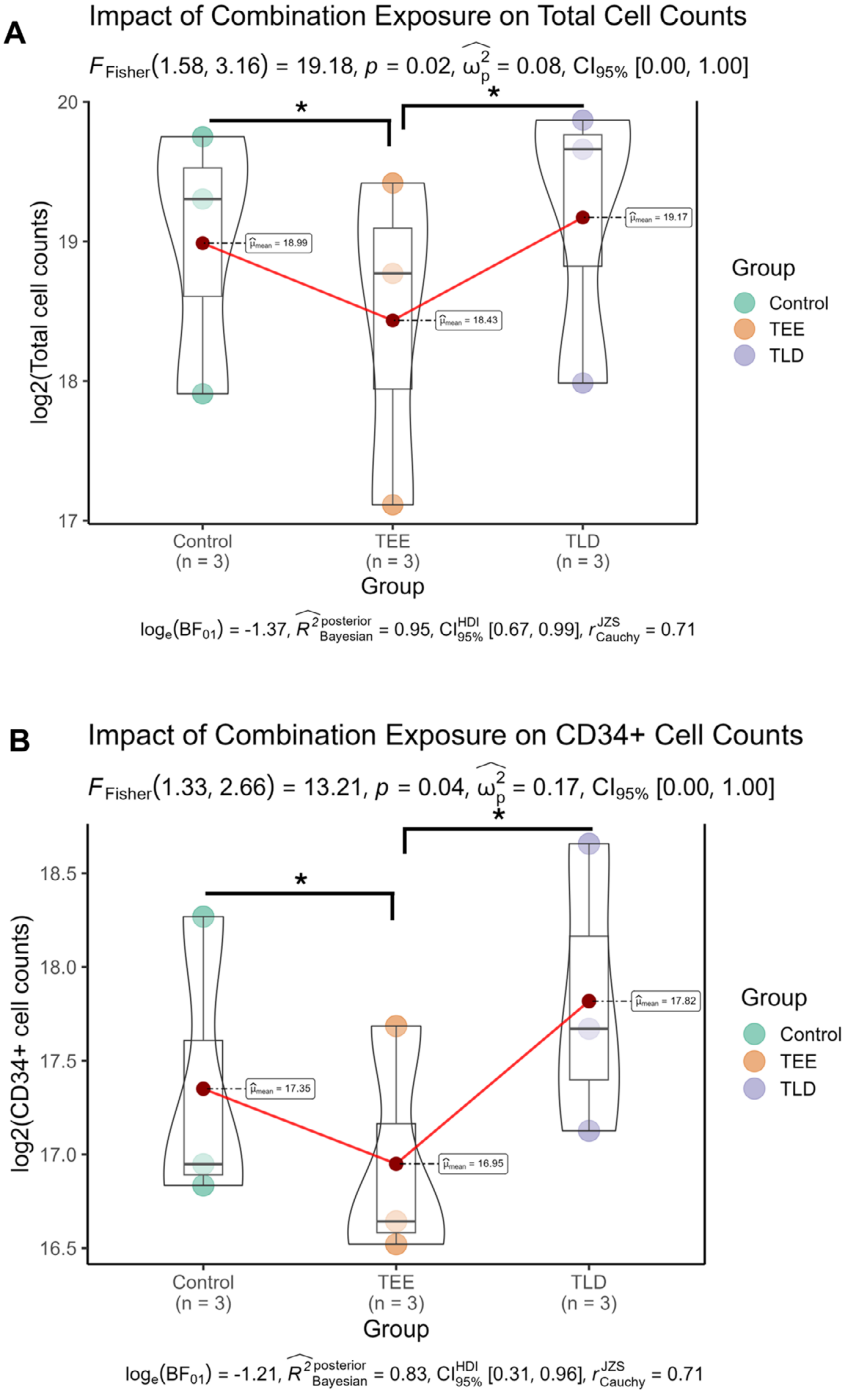
Total mononuclear and CD34+ cell counts after 7‐day culture in the presence of drug combinations. Violin with a nested box and whisker plots showing log2 transformed cell counts for total (A) and CD34+ enriched (B) populations. In both cases, statistically significant decreases were noted in TEE exposed groups compared to both paired control and TLD‐exposed groups. Data is representative of three independent biological replicates.

After experiments with multiple concentrations had been completed, cell count and immunophenotype after a 7‐day culture period were undertaken. Violin with nested box and whisker plots showing log2 transformed cell counts for total viable cells and CD34+ cells are shown in Figure 2A,B. Total and CD34+ counts were significantly lower with TEE (*p* ≤ 0.05) when compared to VC and TLD.

**FIGURE 3:**
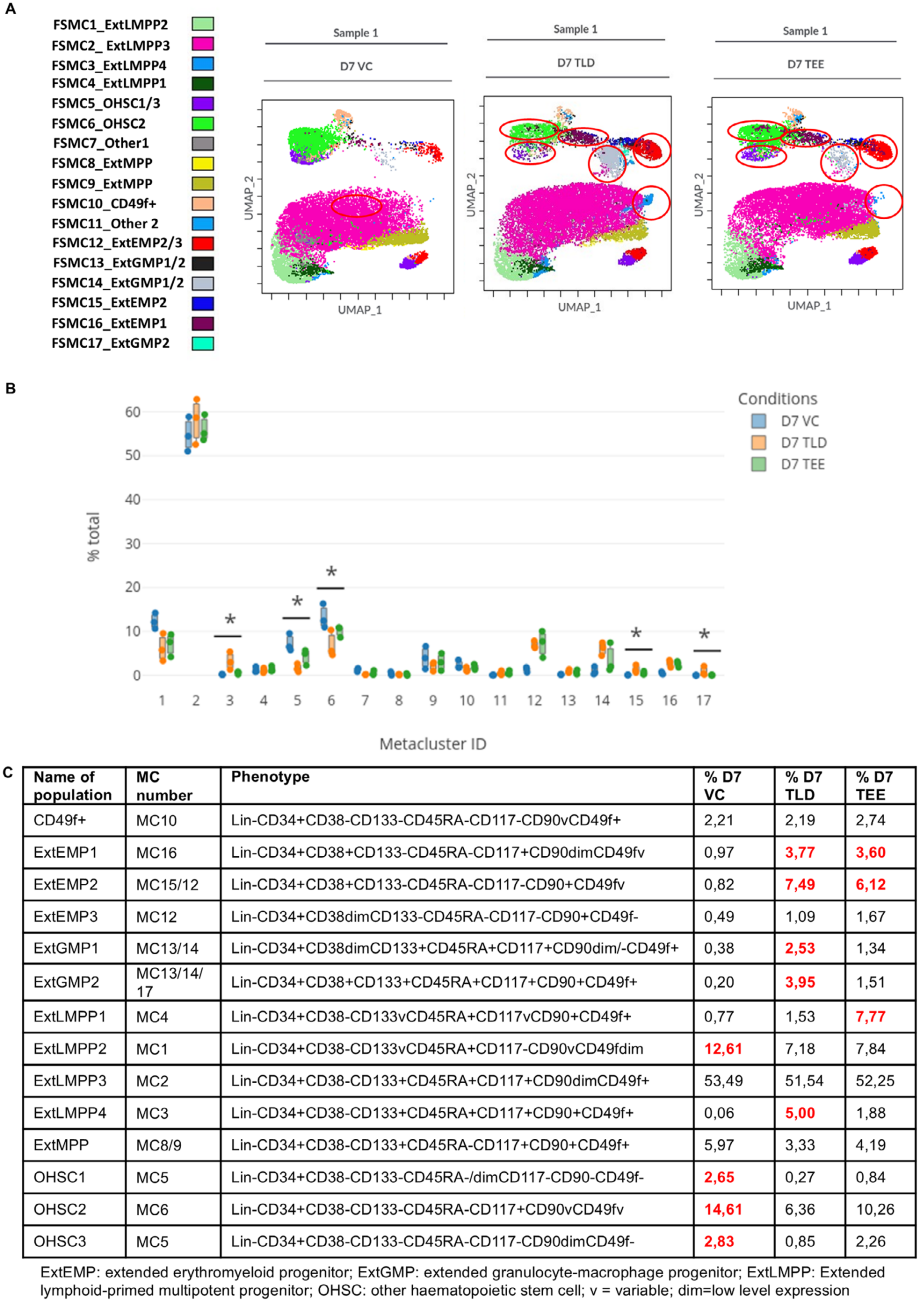
Immunophenotypic analysis of HSPCs after 7‐day growth in the presence of combination ARVs. (A) UMAP FlowSOM MC dot overlay plot showing the MCs coloured according to the scale shown on the left. 17 numbered MCs are shown with the corresponding named sub‐population adjacent to it. All drug combinations are shown for only one sample. MC15 corresponds to ExtEMP2 and MC17 to ExtGMP2. OHSC1 and 3 make up MC5 in the FlowSOM. Changes occuring in the presence of TLD and TEE are circled in red. (B) Box plot showing the significantly different MCs between the different drug conditions. *p ≤ 0.05. These MCs were identified as MC3, 5, 6, 15, and 17. MC3, 15, and 17 were greater in TLD and MC5 and 6 in VC. (C) Table depicting the immunophenotypic sub‐populations present on D7 with relative percentages of each in the different drug combinations (*n* = 3 for each drug condition) as well as the corresponding metacluster number. Immunophenotypic sub‐populations were gated on the UMAP according to the FlowSOM MCs. Phenotypic sub‐population names are in the first column, followed by the metacluster number, the phenotype and the relative mean percentages observed in the VC and drug conditions on D7. Changes observed between the VC and drug conditions are highlighted in red (*n* = 3)

Concatenated UMAP images for all drug conditions are shown in Figure S2A. The FlowSOM‐guided MCs are shown in Figure 3A together with the population name attached to the MC. Manual gating of the UMAP to obtain the sub‐populations is shown in Figure S2A and the phenotypes and mean percentage of each sub‐population, from three biological repeats, are depicted in Figure 3C. The overlaid UMAP is shown in Figure S2B. From the phenotype table (Figure 3C), the following observations were made: all drug conditions had a predominance of an extended lymphoid‐primed multipotent progenitor 3 [lympho‐myeloid primed progenitors] (ExtLMPP3, MC2) population on D7. LMPPs give rise to both lymphocytes and granulocytes and originate from multipotent progenitors (MPP) from which all subtypes can develop [44]. What is noteworthy is that all extended erythromyeloid progenitor (ExtEMP) populations, which give rise to both megakaryocyte‐erythrocyte progenitors (MEP) and eosinophils/basophils [44], were higher in the presence of drugs (TLD and TEE) compared to the VC. The most marked difference was in ExtEMP2 making up 0.82%, 7.49%, and 6.12% for VC, TLD, and TEE, respectively. The same was seen for extended granulocyte‐macrophage (granulocyte‐monocyte) progenitors (ExtGMP), from which neutrophils and macrophages develop, with a frequency for ExtGMP2 of 0.2%, 3.95%, and 1.51% for VC, TLD, and TEE, respectively. Besides these known populations, three populations not previously described and denoted as other haematopoietic stem cell (OHSC) 1–3 were identified and were higher in the VC. These populations were all primitive as informed by the Lin‐CD34+CD38‐CD133‐CD45RA‐ phenotype.

Statistical analysis revealed five significantly different (*p* < 0.05) MCs, namely, MC3, 15, and 17, which were present more frequently in TLD, and MC5 and 6, which were more prominent in the VC (Figure 3B). Phenotypic correlation of heatmaps and histograms (Figure S3B) with the phenotypic table in Figure 3C showed that MC3 corresponds to ExtLMPP4, MC15 to ExtEMP2, and MC17 to ExtGMP2. For MC5, there were two clusters noted on the FlowSOM map corresponding to OHSC1 and OHSC3, while MC6 corresponds to OHSC2. All these sub‐populations (ExtLMPP4, ExtEMP2, ExtGMP2) were also more frequent in TLD in Table 1 while OHSC1, 2, and 3 were more frequent in VC.

**TABLE 1:** Immunophenotype of colony forming units based on UMAP gating on D14.

Name of population	Phenotype	AVG % D14 VC	AVG % D14 TLD	AVG % D14 TEE
RBC1	CD235a + CD71 + CD41 − CD33 − CD14 − CD15−	8.62	**0.04**	10.99
RBC2	CD235a + CD71 + CD41 − CD33 − CD14 − CD15−	2.81	3.16	3.62
RBC3	CD235a + CD71 + CD41 − CD33 − CD14 − CD15 − CD56 + myeloid+	0.36	0.01	0.58
Neut1	CD235a − CD71vCD41 − CD33 + CD14 − /dimCD15+	9.35	**32.49**	6.26
Neut2	CD235a − CD71 − CD41 − CD33 − CD14 − CD15+	3.59	1.37	1.91
Neut3	CD235a + CD71 + CD41 − CD33dimCD14 − CD15dim	4.62	1.19	2.58
Myeloid 5	CD235a + CD71 + CD33 + CD14 − CD15−	7.21	7.63	6.30
CD56 + Myeloid	CD235a − CD71dimCD41 − CD33 + CD14 + CD15dim	3.02	4.57	2.04
Myeloid 2	CD235a − CD71 + CD41 − CD33 − CD56 + myeloid+CD14 − CD15dim	0.74	0.32	0.65
Myeloid 3	CD235a − CD71 − CD41 − CD33 + CD14 + CD15v	46.03	42.94	51.96
Myeloid 4	CD235a − CD71 − CD41vCD33+ CD56 + myeloid+CD14 − CD15dim	1.96	0.69	1.32
Plt	CD235a − CD71 − CD41 + CD33 + CD14 + CD15v	7.60	2.10	8.71

*Note:* The above table shows the immunophenotypic sub‐populations present within the CFU colonies after 14 days in culture with the different drug combinations (TLD and TEE) compared to the control. Neutrophil colonies are markedly increased in the presence of TLD compared to TEE and the VC. RBC1 and 3 are near absent in the presence of TLD, in keeping with what was observed on colony counting. Marked changes in the presence of TLD are denoted in red.

Abbreviations: dim = low level expression; myeloid = myeloid population; neut = neutrophil population; Plt = platelet population; RBC = red blood cell population; TEE = tenofovir disoproxil fumarate, emtricitabine, and efavirenz; TLD = tenofovir disoproxil fumarate, lamivudine, and dolutegravir; v = variable expression; VC = vehicle control.

**FIGURE 4:**
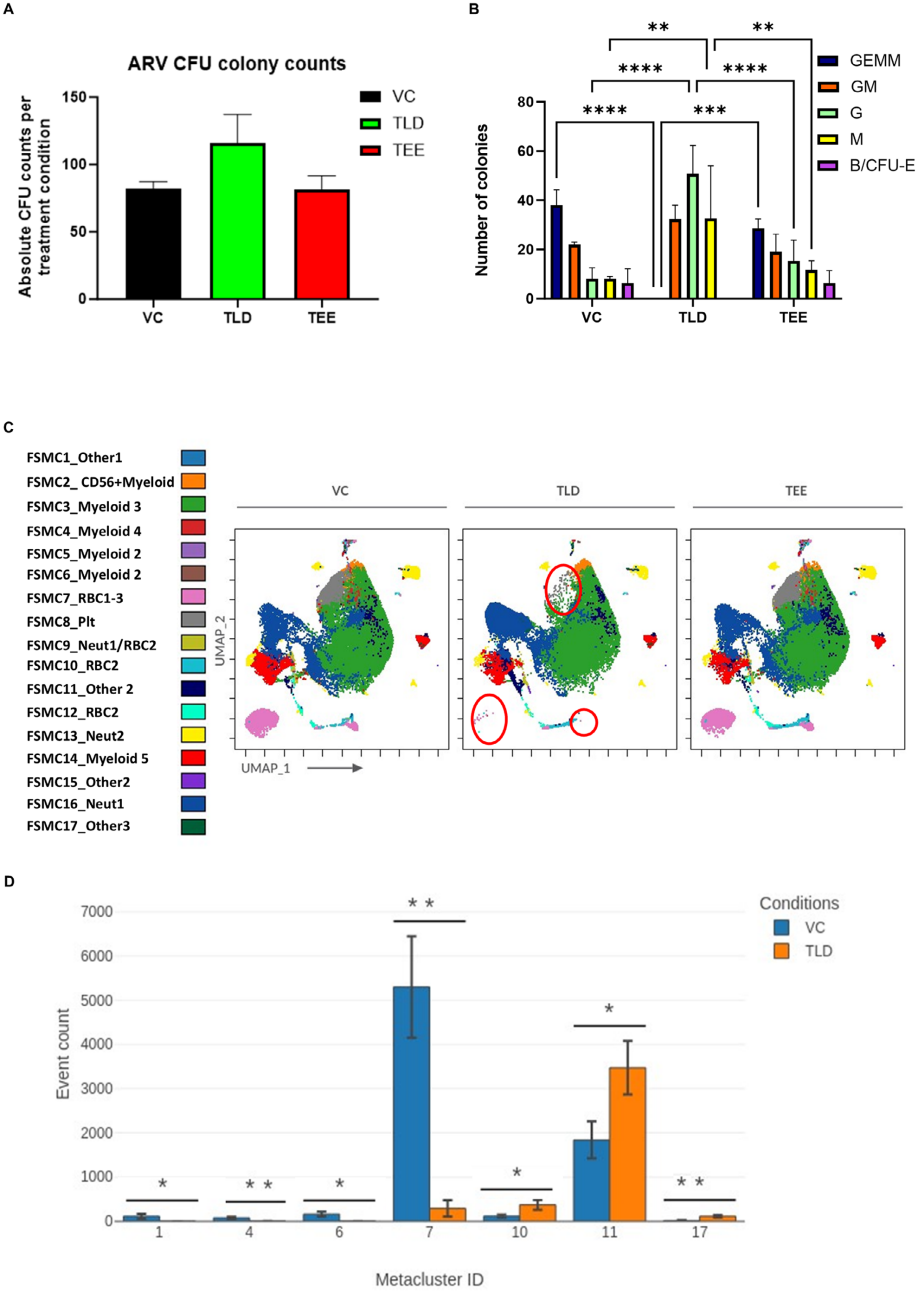
CFU immunophenotype after 14 days exposure to ARVs. (A) Bar graph depicting the total number of colony forming units identified between the VC as well as the other ARV combinations. Average number of colonies for VC is 82, for TLD 115 and for TEE 81. (B) Bar graph depicting the number and types of colonies identified with TLD and TEE versus control on D14. TLD had the highest average number of colonies overall with CFU‐G and CFU‐M being statistically significantly more than TEE and VC (****p < 0.0001) and (**p < 0.01), respectively. No CFU‐GEMM or B/CFU‐E were found with TLD (*n* = 3). (C) FlowSOM dot overlay map from original UMAP shown in Figure S4A–C, coloured according to the MCs on the left and displaying all drug conditions. Changes occuring in the presence of TLD are circled in red. (D) Bar graph showing the statistically significant MCs (1, 4, 6, 7, 10, 11, and 17) present between VC (blue bar) and TLD (orange bar). No statistically significant MCs were present between the VC and TEE. The MC identity is on the x‐axis, while the event count is depicted on the y‐axis. *p ≤ 0.05, **p ≤ 0.01.

CFU analysis after culture with TLD and TEE for 14 days showed that the average number of total colonies produced by the VC, TLD, and TEE was 82, 115, and 81, respectively (Figure 4A). In identifying the colony subtypes, TLD showed statistically significantly more CFU‐G (*p* < 0.0001) and CFU‐M (*p* < 0.01) than the VC and TEE but did not contain a single CFU‐GEMM or B/CFU‐E (Figure 4B). After colony recognition, the immunophenotypic analysis was performed using a UMAP as shown in Figure S4A. Gating (Figure S4B,C) was guided by FlowSOM MCs (Figure 4C, with population names), followed by overlaying gated populations onto the UMAP image (Figure S5A). What is most notable in the UMAP images is the near absence of RBC1 and RBC3 with TLD. Phenotypes and associated mean percentages of sub‐populations for drug conditions are shown in Table 1. The identified CD56+ myeloid population is believed to be monocytes expressing CD56 [45], as this population is brightly positive for CD33 and CD14. With TLD, CD235a (Glycophorin A receptor) [46] and CD71 (transferrin receptor) [46] denoting erythroid‐containing cells [47] were lower (see bivariate plot in Figure S5B), while CD33/CD15 co‐expressing populations (see Table 1), denoting neutrophils [48], were higher.

Seven CFU MCs were found to be significantly different between the VC and TLD, namely MC1, 4, 6, 7, 10, 11, and 17, but none between VC and TEE (Figure 4D). The heatmaps better portraying the phenotype of these MCs are shown in Figure S5C. MC7 is the dual‐expressing CD235a/CD71 population, correlating to RBC1‐3, confirming a significant difference (**p ≤ 0.01) in RBC populations between TLD and the VC. MC11 expresses CD33, CD14, and CD15, indicating a myeloid population, but was not gated separately as it was overlying another cluster. MC1, 4, and 6 are all present at a higher frequency in the VC compared to TLD, but the event counts and thus clusters are almost indistinguishable from one another. They are, however, all brightly positive for CD235a, confirming that these are RBC populations.

## 4 Discussion

This study explored the impact of ARV exposure on UCB‐derived HSPCs as an in vitro model of potential in utero exposure. We found that EFV increased cytotoxicity and reduced cell metabolic activity in acute and chronic exposures even at 0.5× *C*_max_, which has been shown to be the level of exposure in M:C ratios clinically [10]. When added in combination with other drugs in TEE (EFV, FTC, and TDF), similar findings were observed, and significantly lower CD34+ cell counts were found in the presence of EFV compared to TLD and the VC after 7 days. In clinical studies, EFV has been found to have a very good overall safety and efficacy profile [15], with some evidence of in utero neurotoxicity [49], teratogenicity [50], hepatotoxicity [51], and neutropenia [52]. The latter has not been described in HEU infants. However, an interesting in vitro study of the effect of EFV on hepatic cells showed similarly that viability and cell proliferation were impaired within 24 h of exposure and that the effect was dose‐dependent [53]. The authors concluded that these effects were secondary to acute mitochondrial toxicity, including increased production of superoxide, amongst others, and that similar effects may be observed in other cell lines.

Unexpectedly, DTG and TLD were found to stimulate HSPC growth at certain concentrations, with increased cellular metabolic activity at these doses after 7‐day exposure. The cause of this effect could not be established. The CFU data, however, show that the presence of TLD inhibits the formation of CFU‐GEMM and B/CFU‐E colonies within MethoCult after 14 days in culture. This was illustrated by the absence of these colonies on physical colony counting and confirmed with CFU immunophenotyping. The difference between D7 and D14 findings may be that StemSpan AOF medium (liquid) and MethoCult (semi‐solid) are not identical in their make‐up or cytokine composition [54, 55]. Variations in protein content and matrix properties between these media could influence drug‐protein interactions, potentially altering the in vitro exposure and resulting in a technical artefact. An alternative explanation may be that one of the drugs in the combination actually caused a direct inhibition of erythrocyte development. Since TDF was present in the other drug combination (TEE), it is unlikely to be the responsible agent. Of the two remaining drugs, DTG is not known to cause major haematological abnormalities, with side effects in this system limited to isolated case studies [56, 57]. DTG has also been shown to improve anaemia and leucopenia in adults LWH [58]. 3TC, on the other hand, has been implicated in the development of pure red cell aplasia (PRCA) in a number of reports [59, 60]. PRCA causes a profound normocytic anaemia secondary to inhibition of erythrocyte precursors in the bone marrow [61]. The absence of similar findings with CFUs in the TEE regimen is intriguing considering the very similar molecular structures of 3TC and FTC [62], both of which are cytidine analogues, and the fact that similar findings of FTC inducing PRCA have been reported [63, 64]. One possible explanation may be that 3TC has a significantly greater affinity for the mitochondrial DNA polymerase gamma enzyme, leading to mitochondrial dysfunction, which is known to be the mechanism responsible for nucleoside analogue toxicity [65, 66]. Mitochondrial dysfunction can lead to anaemia, as heme biosynthesis in erythroid precursors relies on mitochondria [67]. Despite this potential toxicity, mitochondrial toxicity secondary to 3TC has rarely been reported [68].

In summary, we observed significant HSPC cytotoxicity secondary to EFV and TEE following both acute (24 h) and chronic (7‐day) exposure. This level of haematological toxicity with EFV has not previously been reported and provides evidence for potentially adverse haematological events seen in HEU neonates. This should be borne in mind in HEU infants whose mothers took an EFV‐containing regimen during pregnancy. DTG stimulates Lin‐CD34+ HSPCs in culture, although the mechanism is not understood. Finally, TLD inhibits the formation of CFU‐GEMM and B/CFU‐E in vitro. A potential reason may be that 3TC induces mitochondrial dysfunction in erythroid precursors, but this cannot be accurately determined from our data. This study provides evidence for an array of in vitro toxicities following exposure to ARV drugs at clinically relevant doses that HEU infants are likely to be exposed to in vivo *and* highlights the need for research into the potential complications in this vulnerable population secondary to in utero ARV exposure. In our HIV‐endemic population in South Africa, this is particularly important to explore given our previous argument supporting the inclusion of UCB from HEU infants in haematopoietic stem cell transplantation [69].

## 5 Limitations

The presence of an antibody to CD10 in our immunophenotype panel would have allowed for better discrimination of population subsets. In addition, this study did not include an in vivo component, and we therefore cannot determine with certainty whether these changes are clinically translatable.

## Author Contributions

**Candice Laverne Hendricks:** conceptualization (equal), data curation (equal), formal analysis (lead), investigation (lead), methodology (equal), writing – original draft (lead), writing – review and editing (lead). **Andrea Ellero:** conceptualization (equal), data curation (equal), formal analysis (equal), investigation (equal), methodology (equal), validation (equal), writing – review and editing (equal). **Juanita Mellet:** conceptualization (equal), investigation (supporting), methodology (supporting), supervision (equal), writing – review and editing (equal). **Voula Stivaktas:** conceptualization (equal), data curation (equal), formal analysis (equal), writing – review and editing (equal). **Michael Sean Pepper:** conceptualization (equal), funding acquisition (lead), methodology (supporting), supervision (equal), writing – review and editing (equal).

## Ethics Statement

Approval was obtained from the University of Pretoria, Faculty of Health Sciences Research Ethics Committee, study number 102/2020. Enrolled mothers gave written informed consent before undergoing a caesarean section for the collection of umbilical cord blood for the study following the delivery of their infants.

## Conflicts of Interest

M.S.P. has received research grants from the South African Medical Research Council, the Bill and Melinda Gates Foundation, the South African National Health Laboratory Service Research Trust, The National Research Foundation (South Africa) and the Wellcome Trust. M.S.P. is a co‐founder and non‐remunerated Board Member of Antion Biosciences and Altera Biosciences. C.L.H. is a non‐remunerated board member of DKMS Africa, a not‐for‐profit organisation.

## Supporting information


**Figure S1.** Effect of LDH release upon acute drug exposures.
**Figure S2.** Lin‐CD34+ Immunophenotypic sub‐populations on D7.
**Figure S3.** Identification of the significantly different Lin‐CD34+ MC phenotypes after.
**Figure S4.** Colony forming unit immunophenotypic sub‐populations.
**Figure S5.** Differences in immunophenotypic sub‐populations identified between TLD.

## Data Availability

For original flow cytometry data files, please contact michael.pepper@up.ac.za.
