# Supplementary material for: Antiretroviral‐Induced Toxicity in Umbilical Cord Blood‐Derived Haematopoietic Stem/Progenitor Cells
Source: J Cell Mol Med. 2025 Apr 27;29(8):e70557. doi: 10.1111/jcmm.70557 (PMC12034851; doi:10.1111/jcmm.70557)

Antiretroviral induced toxicity in umbilical cord blood-derived haematopoietic stem/progenitor cells

Supplementary data figure legends

Figure S1: Effect of LDH release upon acute drug exposures

Violin with nested box and whisker plots are used for all figures. LDH release after 24 hours of drug exposure to 3TC, DTG, FTC and TDF is portrayed on the y-axis and the concentration of drug on the x-axis: 3TC (A) DTG (B), TDF (C), FTC (D) shown. Each coloured dot represents one technical repeat for each concentration. No significant increase in LDH release is seen compared to the control for all drugs at all concentrations. n=9 refers to 3 biological replicates in triplicate.

Figure S2: Lin-CD34+ Immunophenotypic sub-populations on D7

A. UMAP plots for VC, TLD and TEE shown. Images are concatenated for 3 biological replicates (n=3). Colouring scale is present on the right of each colouring channel and the marker for the channel is presented at the top. Only CD38, CD133 and CD45RA are shown for ease of visualisation. The manually gated and named sub-populations are shown directly on the UMAP. These manual gates were guided by the FlowSOM metacluster dot overlay plots shown in Fig. 3A. Nomenclature is as per the table in Fig. 3C.
B. UMAP plots showing the backgated immunophenotypic sub-populations which were manually gated as shown in A. Sub-population nomenclature is shown on the coloured scale on the left of the image. The VC as well as TLD and TEE are shown. ExtLMPP3 is the dominant population in all conditions. ExtLMPP4 is nearly absent in the VC, while OHSC1-3 is most prominent in the VC. ExtEMP1-3 and ExtGMP1 and 2 have a higher density in TLD. Only one of the three UCB replicates is shown but the Cytobank platform analysis was performed on all three individual UCB samples separately.

Figure S3: Identification of the significantly different Lin-CD34+ MC phenotypes after 7 days in culture

A. Heatmaps for VC, TLD and TEE are shown which describe the immunophenotype of the MC found to be significantly different between conditions as seen in Fig. 3B. Only one sample is shown for each drug condition.

B. The corresponding histograms are shown for VC and TEE to correlate the phenotype in the heatmaps as well as in the table in Fig. 3C.

Figure S4: Colony forming unit immunophenotypic sub-populations

A. UMAP images of the CFU immunophenotypes for all conditions. UMAPs are coloured according to level of expression indicated on the vertical scale at the right of each colouring channel. Colouring channel labels are indicated on the top and are the z-scale. 3 individual biological replicates were concatenated for each image subset (VC, TLD, TEE). Noteworthy differences are the markedly decreased CD235a and CD71 expressing populations in TLD compared to TEE and VC. CD41+ cells are also CD33+ and likely indicate platelet binding on the myeloid cells. TLD appears to have a larger populations expressing CD33, CD14 and CD15. Population frequencies are shown in Table 2.
B. UMAP images of one sample of the VC is depicted to show the gating that was employed to identify the different immunophenotypic sub-populations. Colouring channels on the z-axis is presented above each UMAP plot (only CD235a, CD71, CD41 and CD33 are shown) and the scale of expression is presented on the right. Manual gates were guided by the FlowSOM metacluster dot overlay plots in Fig. 4C.

Figure S5: Differences in Immunophenotypic sub-populations identified between TLD, TEE and VC on colony forming unit analysis after 14 days in culture

A. The backgated (overlaid) UMAP image is shown where the MC identified during the FlowSOM were gated and given population names according to the immunophenotype present. RBC1 and 3 is near absent and Neut1 more prominent with TLD. Only one sample is shown for easier visualisation. See also frequencies of populations in Table 2.
B. Bivariate plot coloured according to the populations on the left of the figure and showing CD235a expression on the y-axis and CD71 expression on the x-axis for each drug condition. RBC1 and 3 (same MC) are CD235a and CD71 dual expressing and are absent in TLD. CD71 expression is, however, still present with TLD. Analysis was performed on 3 separate UCB samples.
C. Heatmaps of the statistically significant MCs are displayed to identify the immunophenotype of each MC and correlate it to the phenotypes in Table 2. MC 1, 4, and 6 were higher in the VC and present at very low event counts. They thus did not make up distinct sub-populations that could be identified, but all expressed CD235a brightly, which is in keeping with the RBC predominance in the VC compared to TLD.

MC 6 and 7 had a similar phenotype in keeping with RBC1, once again confirming the paucity of RBC populations in TLD. MC 11 was not distinguishable on the UMAP as a separate MC but rather was underlying MC 13 and 14. It did, however, have all the myeloid markers (CD33, CD14, CD15) and was phenotypically similar to the CD56 myeloid population, which was present at a higher frequency in TLD. Finally, MC 17 also had too low an event count to be a separate MC, however, it represented a neutrophil population (CD15 bright +) and was greater in the TLD population. See also Fig. 4D.

Figure S1: Effect of LDH release upon acute drug exposures

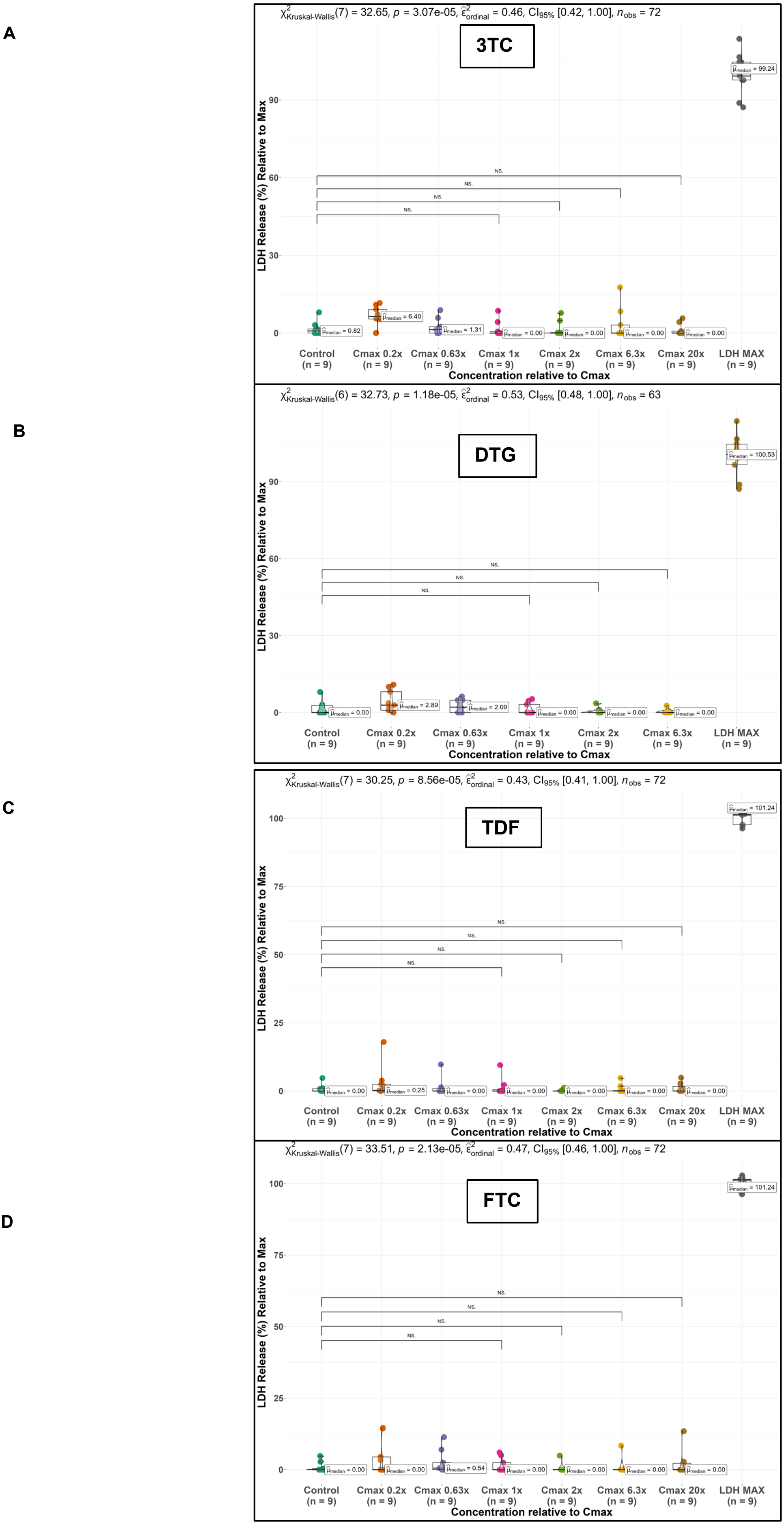

Figure S2: Lin-CD34+ Immunophenotypic sub-populations on D7

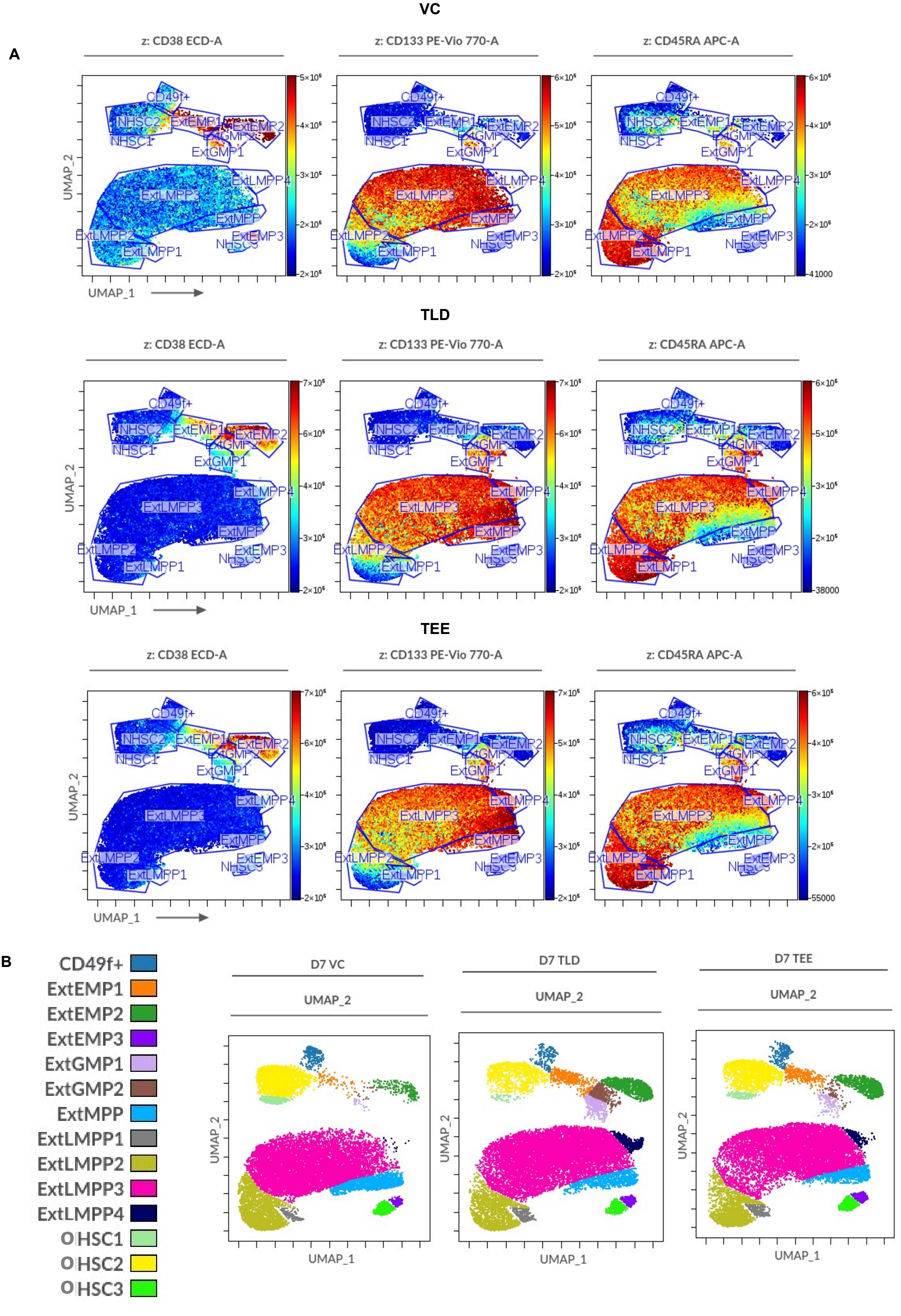

Figure S3: Identification of the significantly different Lin-CD34+ MC phenotypes after 7 days in culture

A

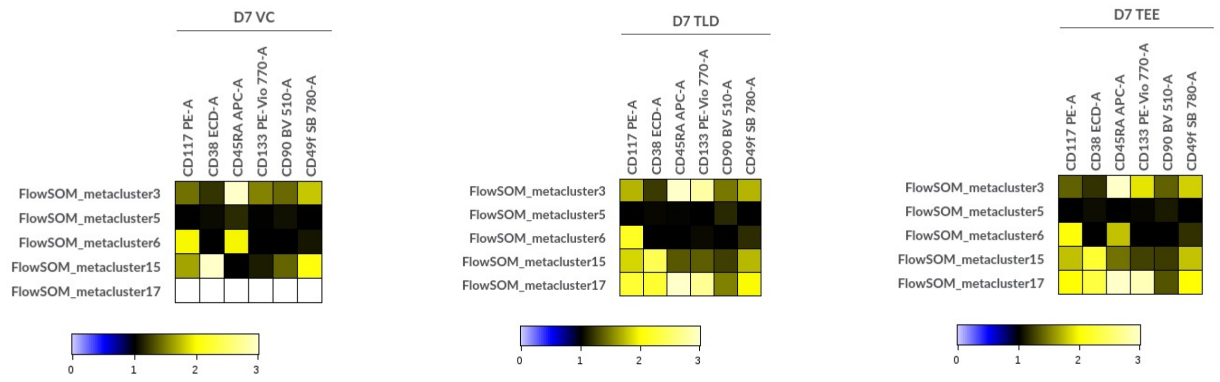

B

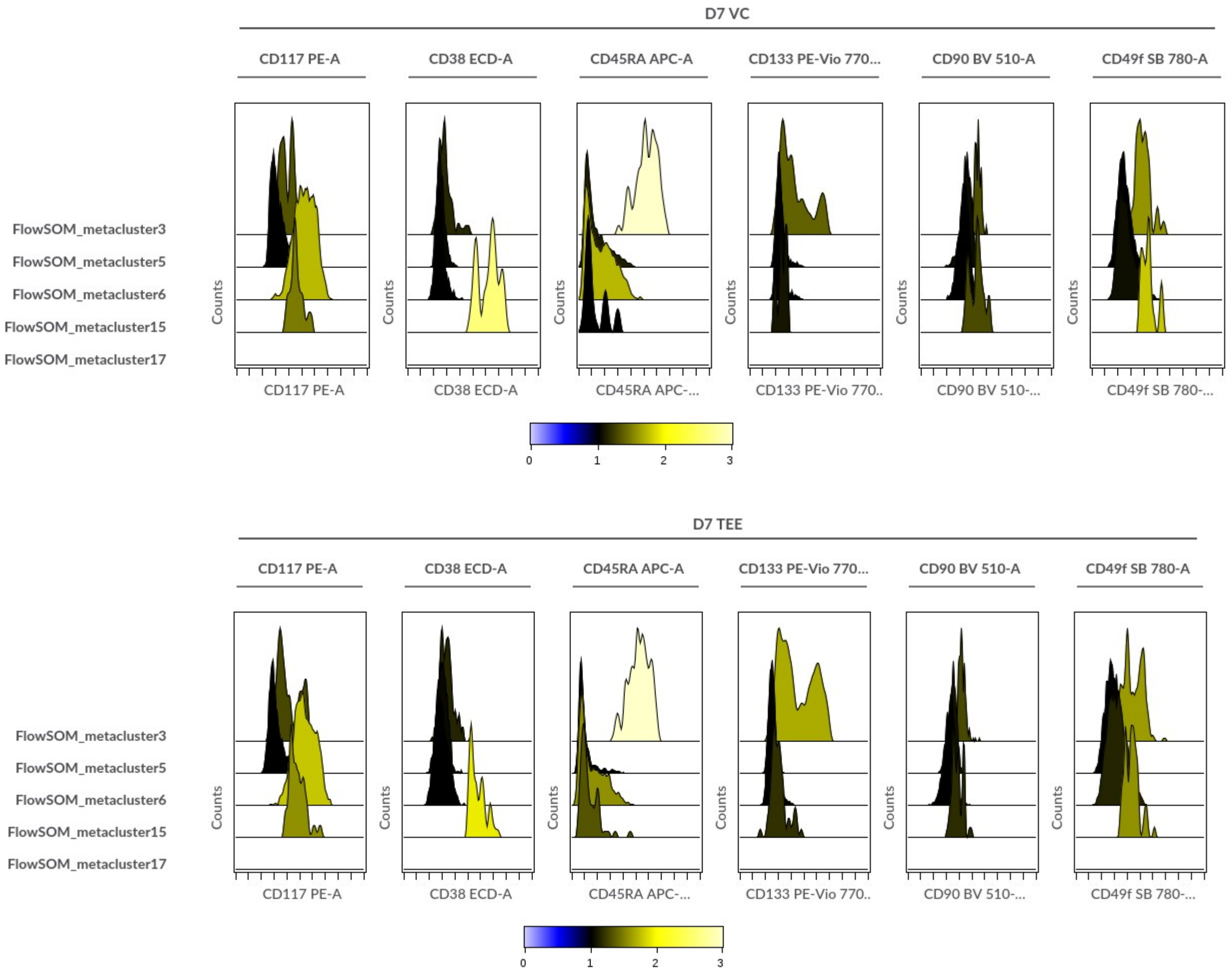

Figure S4: Colony forming unit immunophenotypic sub-populations

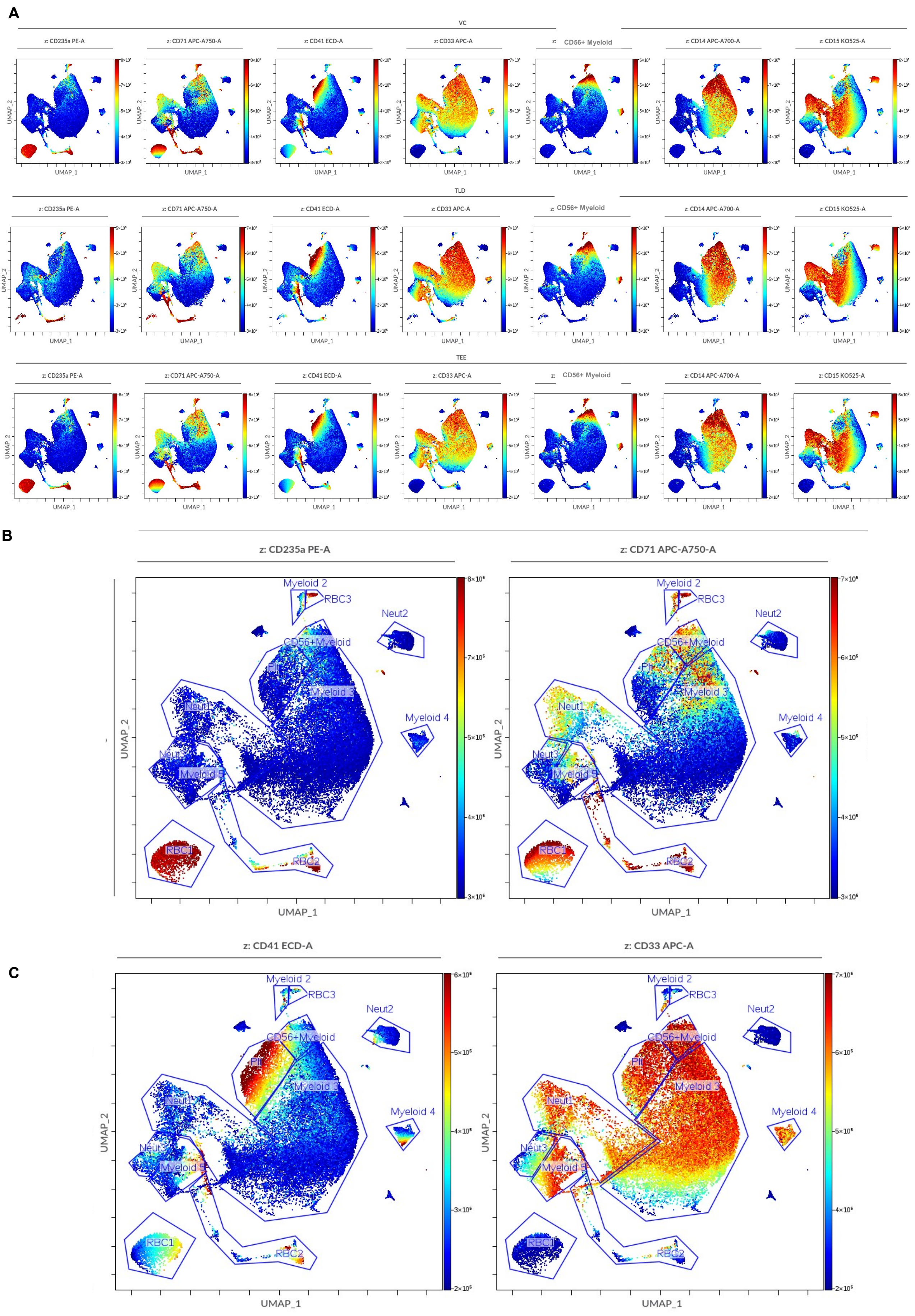

Figure S5: Differences in Immunophenotypic sub-populations identified between TLD, TEE and VC on colony forming unit analysis after 14 days in culture

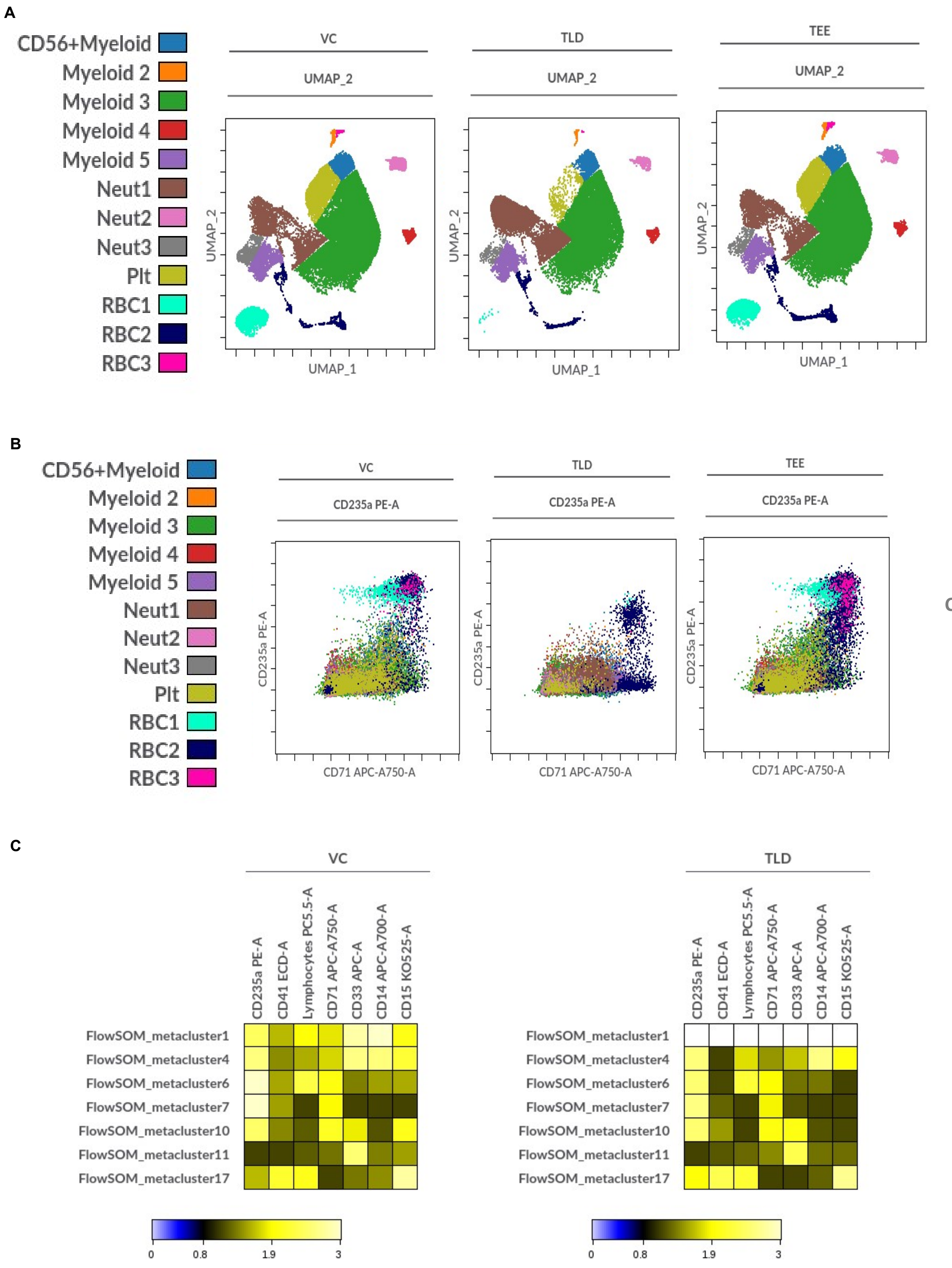

Supplement: Supplementary file 1 — Figure S1. Effect of LDH release upon acute drug exposures. Figure S2. Lin‐CD34+ Immunophenotypic sub‐populations on D7. Figure S3. Identification of the significantly different Lin‐CD34+ MC phenotypes after. Figure S4. Colony forming unit immunophenotypic sub‐populations. Figure S5. Differences in immunophenotypic sub‐populations identified between TLD. [file JCMM-29-e70557-s001.pdf]
